# Supplementary material for: Building an RNA-Based Toggle Switch Using Inhibitory RNA Aptamers
Source: ACS Synth Biol. 2022 Feb 8;11(2):562–9. doi: 10.1021/acssynbio.1c00580 (PMC9007568; doi:10.1021/acssynbio.1c00580)
Supplement: Supplementary file 1 — sb1c00580_si_002.pdf [file sb1c00580_si_002.pdf]

Supporting Information
Building an RNA-based Toggle Switch using Inhibitory RNA Aptamers

Alicia Climent-Catala,[†,‡] Thomas E. Ouldridge,[†,¶] Guy-Bart V. Stan,[†,¶] and Wooli Bae*[,†,¶,§]

†*Imperial College Centre for Synthetic Biology, London, SW7 2AZ, U.K.*

‡*Department of Chemistry, Imperial College London, London, SW7 2AZ, U.K.*

¶*Department of Bioengineering, Imperial College London, London, SW7 2AZ, U.K.*

§*Current address: Department of Physics, University of Surrey, Guildford, GU2 7XH, U.K.*

E-mail: w.bae@surrey.ac.uk

SI Table 1: DNA sequences used in this work.

Name	Sequence (5'-3')
T7 promoter	TAATACGACTCACTATAG
SP6 promoter	ATTTAGGTGACACTATAG
tBroccoli	GCCCGGATAGCTCAGTCGGTAGAGCAGCGGAGACGGTC GGGTCCAGATATTCGTATCTGTCGAGTAGAGTGTGGGC TCCGCGGGTCCAGGGTTCAAGTCCCTGTTCGGGC
Malachite Green	GGATCCCGACTGGCGAGAGCCAGGTAACGAATGGATCC
CChMVd-U10	GGAAGAGGTCGGCACCTGACGTCGGTGTCCTGA TGATGATCCATGAGAGGATCGAAACCTCTTCTAG
T7 aptamer T230-39[a]	AGGCGAGCGTAAGTCAATTCCACTATCATTGCTGCAAGC
SP6 aptamer mut9[b]	GGAGAGTTGCTTGGAATGCGTTATAGTCTCTTAGGTGTG TTCGCACACCACTCTCC

[a] Sequence is taken from Ohuchi et al. [2012] [b] Sequence is taken from Mori et al. [2012]

SI Table 2: DNA kleptamers against T7 inhibitory aptamer

Name	Sequence (5'-3')
T7 kleptamer 32 nt[a]	GAAGCAGCAATGATAGTGGAATTGACTTACGC
T7 kleptamer 39 nt	GCTTGCAGCAATGATAGTGGAATTGACTTACGCTCGCCT
T7 kleptamer 34 nt	CAGCAATGATAGTGGAATTGACTTACGCTCGCCT
T7 kleptamer 44 nt	GCTTGCAGCAATGATAGTGGAATTGACTTACGCTCGCCT **CTAGA**[b]
T7 kleptamer 44 nt	**CTTCC**GCTTGCAGCAATGATAGTGGAATTGACTTACGCT CGCCT[b]
T7 kleptamer 49 nt	GCTTGCAGCAATGATAGTGGAATTGACTTACGCTCGCCT **CTAGAAGAGG**[b]

[a] Sequence is taken from Lloyd et al. [2018] [b] Toehold sequences in bold

SI Table 3: DNA kleptamers against SP6 inhibitory aptamer

Name	Sequence (5'-3')
SP6 kleptamer 38 nt[a]	TAAGAGACTATAACGCATTCCAAGCAACTCTCCGCTGC
SP6 kleptamer 56 nt	GGAGAGTGGTGTGCGAACACACCTAAGAGACTATAAC GCATTCCAAGCAACTCTCCC
SP6 kleptamer 51 nt	GTGGTGTGCGAACACACCTAAGAGACTATAACGCATT CCAAGCAACTCTCC
SP6 kleptamer 62 nt	**TCTTCC**GGAGAGTGGTGTGCGAACACACC TAAGAGACTATAACGCATTCCAAGCAACTCTCC[b]
SP6 kleptamer 61 nt	GGAGAGTGGTGTGCGAACACACCTAAGAGACTATAAC GCATTCCAAGCAACTCTCC**CTAGA**[b]
SP6 kleptamer 66 nt	GGAGAGTGGTGTGCGAACACACCTAAGAGACTATAAC GCATTCCAAGCAACTCTCC**CTAGAAGAGG**[b]

[a] Sequence is taken from Lloyd et al. [2018] [b] Toehold sequences in bold

SI Table 4: gBlocks

**Name**	**Sequence (5'-3')**
T7: T7apt: HHR tBroccoli: T	TAATACGACTCACTATAGAGGCGAGCGTAAGTCAATTCCACTA TCATTGCTGCAAGCGGAAGAGGTCGGCACCTGACGTCGGTGTC CTGATGATGATCCATGAGAGGATCGAAACCTCTTCTAGGCCCG GATAGCTCAGTCGGTAGAGCAGCGGAGACGGTCGGGTCCAGAT ATTCGTATCTGTCGAGTAGAGTGTGGGCTCCGCGGGTCCAGGG TTCAAGTCCCTGTTCGGGCTCACACTGGCTCACCTTCGGGTGG GCCTTTCTGCGTTTATA
T7: tBroccoli: HHR: T7apt: T	TAATACGACTCACTATAGGCCCGGATAGCTCAGTCGGTAGAG CAGCGGAGACGGTCGGGTCCAGATATTCGTATCTGTCGAGTA GAGTGTGGGCTCCGCGGGTCCAGGGTTCAAGTCCCTGTTCGG GCGGAAGAGGTCGGCACCTGACGTCGGTGTCCTGATGATGAT CCATGAGAGGATCGAAACCTCTTCTAGAGGCGAGCGTAAGTC AATTCCACTATCATTGCTGCAAGCTCACACTGGCTCACCTTC GGGTGGGCCTTTCTGCGTTTATA
T7:tBroccoli	TAATACGACTCACTATAGCCCGGATAGCTCAGTCGGTAGAGC AGCGGAGACGGTCGGGTCCAGATATTCGTATCTGTCGAGTAGA GTGTGGGCTCCGCGGGTCCAGGGTTCAAGTCCCTGTTCGGGCA TGCATGCATGAAAAAAAAAACATGCATGCAGTCGGAAGAGGTC GGCACCTGACGTCGGTGTCCTGATGATGATCCATGAGAGGATC GAAACCTCTTCTAG
SP6: SP6apt: HHR : tBroccoli: T	ATTTAGGTGACACTATAGGGAGAGTTGCTTGGAATGCGTTAT AGTCTCTTAGGTGTGTTCGCACACCACTCTCCGGAAGAGGTC GGCACCTGACGTCGGTGTCCTGATGATGATCCATGAGAGGAT CGAAACCTCTTCTAGGCCCGGATAGCTCAGTCGGTAGAGCAG CGGAGACGGTCGGGTCCAGATATTCGTATCTGTCGAGTAGAG TGTGGGCTCCGCGGGTCCAGGGTTCAAGTCCCTGTTCGGGCT CACACTGGCTCACCTTCGGGTGGGCCTTTCTGCGTTTATA

Name	Sequence (5'-3')
SP6: tBroccoli: HHR:SP6apt: T[a]	ATTTAGGTGACACTATAGGCCCGGATAGCTCAGTCGGTAGA GCAGCGGAGACGGTCGGGTCCAGATATTCGTATCTGTCGAGT AGAGTGTGGGCTCCGCGGGTCCAGGGTTCAAGTCCCTGTTCG GGCGGAAGAGGTCGGCACCTGACGTCGGTGTCCTGATGATGA TCCATGAGAGGATCGAAACCTCTTCTAGGGAGAGTTGCTTGG AATGCGTTATAGTCTCTTAGGTGTGTTCGCACACCACTCTCC TCACACTGGCTCACCTTCGGGTGGGCCTTTCTGCGTTTATA
SP6:tBroccoli	ATTTAGGTGACACTATAGGCCCGGATAGCTCAGTCGGTAGAGCA GCGGAACGGTCGGGTCCATCTGAGACGGTCGGGTCCAGATATTC GTATCTGTCGAGTAGAGTGTGGGCTCAGATGTCGAGTAGAGTGT GGGCTCCGCGGGTCCAGGGTTCAAGTCCCTGTTCGGGCGCCAG GAAGAGGTCGGCACCTGACGTCGGTGTCCTGATGATGATCCATG AGAGGATCGAAACCTCTTCTAGACTGGTACGTCCTGCTGATGAG TCCCAAATAGGACGAAACGCGGAAACGCGTCCAGGACTCCACAG TCCGCTCCCATCCTCACACTGGCTCACCTTCGGGTGGGCCTT TCTGCGTTTATA
T7: T7apt: HHR Malachite: T	TAATACGACTCACTATAGAGGCGAGCGTAAGTCAATTCCACTAT CATTGCTGCAAGCGGAAGAGGTCGGCACCTGACGTCGGTGTCCT GATGATGATCCATGAGAGGATCGAAACCTCTTCTAGGGATCCCG ACTGGCGAGAGCCAGGTAACGAATGGATCCTCACACTGGCTCAC CTTCGGGTGGGCCTTTCTGCGTTTATA
T7: Malachite: HHR: T7apt: T[b]	TAATACGACTCACTATAGGGATCCCGACTGGCGAGAGCCAGGTA ACGAATGGATCCGGAAGAGGTCGGCACCTGACGTCGGTGTCCTG ATGATGATCCATGAGAGGATCGAAACCTCTTCTAGAGGCGAGCG TAAGTCAATTCCACTATCATTGCTGCAAGCTCACACTGGCTCAC CTTCGGGTGGGCCTTTCTGCGTTTATA

Name	Sequence (5'-3')
T7: Malachite: T	TAATACGACTCACTATAGGGATCCCGACTGGCGAGAGCCAGGTA ACGAATGGATCCGGAAGAGGTCGGCACCTGACGTCGGTGTCCTG ATGATGATCCATGAGAGGATCGAAACCTCTTCTAGACTGGTACG TCCTGCTGATGAGTCCCAAATAGGACGAAACGCGGAAACGCGTC CAGGACTCCACAGTCCGCTCCCATCCTCACACTGGCTCACCTTC GGGTGGGCCTTTCTGCGTTTATA
SP6: SP6apt: HHR Malachite: T	TTTAGGTGACACTATAGGGAGAGTTGCTTGGAATGCGTTATAGT CTCTTAGGTGTGTTCGCACACCACTCTCCGGAAGAGGTCGGCAC CTGACGTCGGTGTCCTGATGATGATCCATGAGAGGATCGAAACC TCTTCTAGGGATCCCGACTGGCGAGAGCCAGGTAACGAATGGAT CCTCACACTGGCTCACCTTCGGGTGGGCCTTTCTGCGTTTATA
SP6: Malachite: HHR: SP6apt: T	ATTTAGGTGACACTATAGGGATCCCGACTGGCGAGAGCCAGGT AACGAATGGATCCGGAAGAGGTCGGCACCTGACGTCGGTGTCC TGATGATGATCCATGAGAGGATCGAAACCTCTTCTAGGGAGAG TTGCTTGGAATGCGTTATAGTCTCTTAGGTGTGTTCGCACACC ACTCTCCTCACACTGGCTCACCTTCGGGTGGGCCTTTCTGCGT TTATA
SP6: Malachite: T	TAATACGACTCACTATAGGGATCCCGACTGGCGAGAGCCAGGT AACGAATGGATCCGGAAGAGGTCGGCACCTGACGTCGGTGTCC TGATGATGATCCATGAGAGGATCGAAACCTCTTCTAGACTGGT ACGTCCTGCTGATGAGTCCCAAATAGGACGAAACGCGGAAACG CGTCCAGGACTCCACAGTCCGCTCCCATCCTCACACTGGCTCA CCTTCGGGTGGGCCTTTCTGCGTTTATA

[a] This gBlock was used as a template to change to the T7 promoter via PCR.
[b] This gBlock was used as a template to change to the SP6 promoter via PCR.

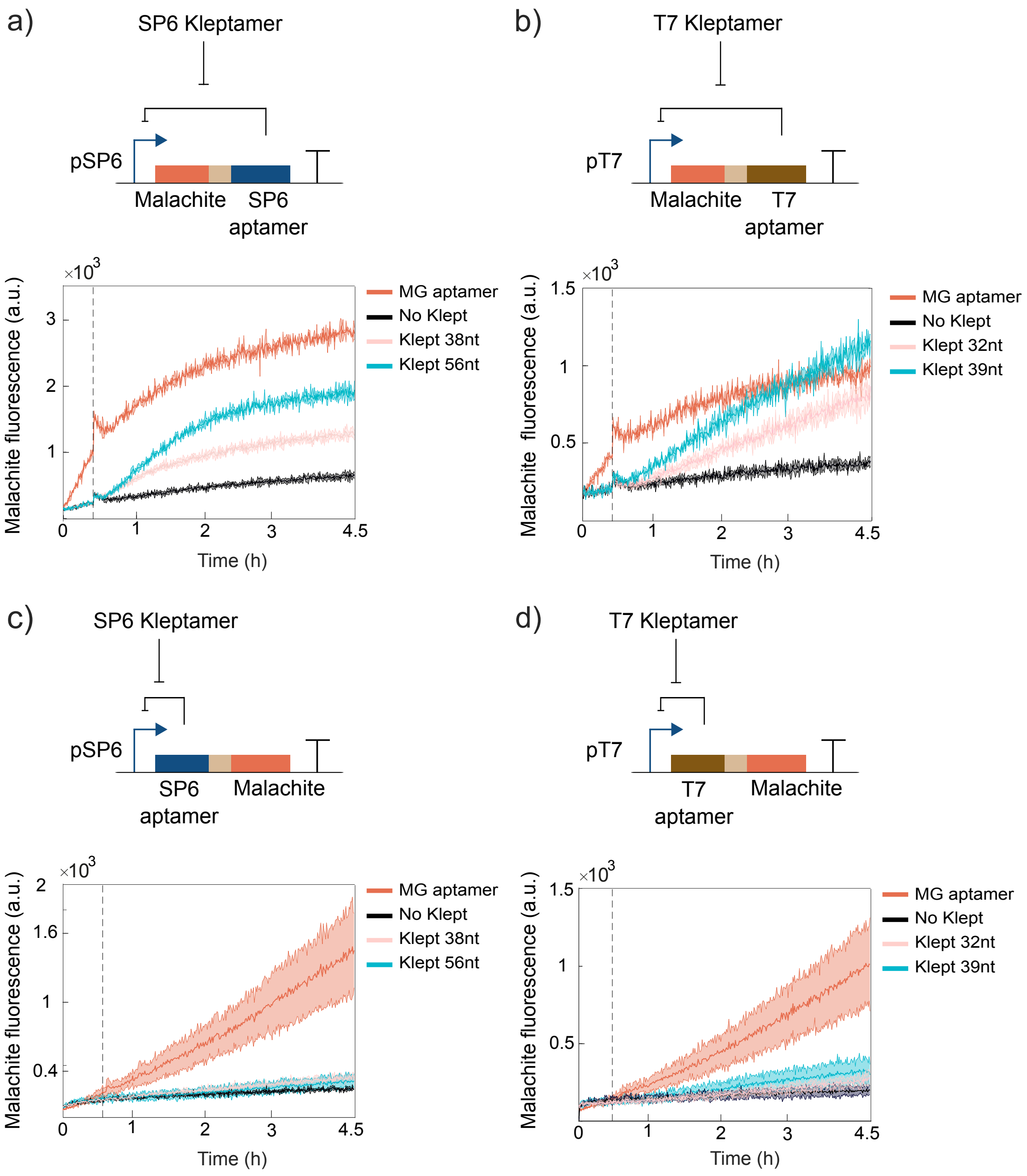

SI Figure 1: **Performance of the T7 and SP6 DNA kleptamers in different architectures.** DNA kleptamers of 38bp for SP6 RNAP and 32bp for T7 RNAP were obtained from Lloyd et al.. Kleptamers of 56bp and 39bp were designed to cover the full sequence of the inhibitory aptamer. a) SP6 cis-acting circuit and b) T7 cis-acting circuit with the fluorescence aptamer being expressed before the inhibitory aptamer. c) SP6 cis-acting circuit and d) T7 cis-acting circuit with the inhibitory aptamer being expressed before the fluorescence aptamer. Spikes in fluorescence near the times indicated by the vertical lines are due to injection-induced temperature decrease. Shaded coloured areas represent the standard deviation over triplicates experiments, and the solid lines within the shaded areas correspond to the average of the fluorescence values.

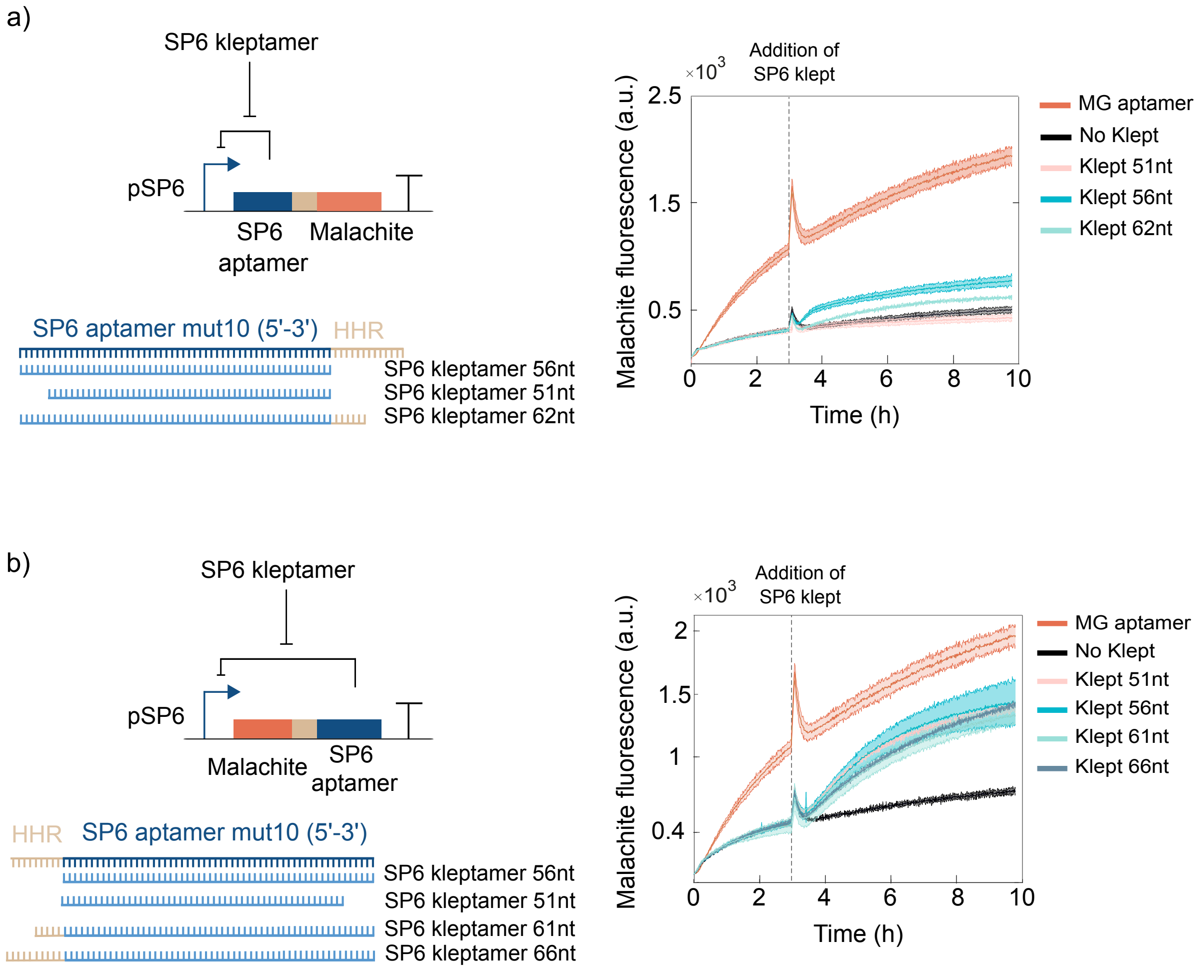

SI Figure 2: **Comparison of the performance of SP6 cis-acting circuits with different architectures with their corresponding kleptamers. a)** Cis-acting circuits where the transcription of the SP6 inhibitory aptamer is followed by the Malachite fluorescent aptamer via SP6 promoter. SP6 kleptamers covering the full and partial inhibitory aptamer were used as well as with a short toehold downstream. **b)** SP6 cis-acting circuits where the transcription of the Malachite Green aptamer is followed by the SP6 inhibitory aptamer. Red lines indicates the expression of only Malachite Green fluorescence signal showing the cost of expressing more elements in the same transcript. Spikes in fluorescence near the times indicated by the vertical lines are due to injection-induced temperature decrease. Shaded coloured areas represent the standard deviation over triplicates experiments, and the solid lines within the shaded areas correspond to the average of the fluorescence values.

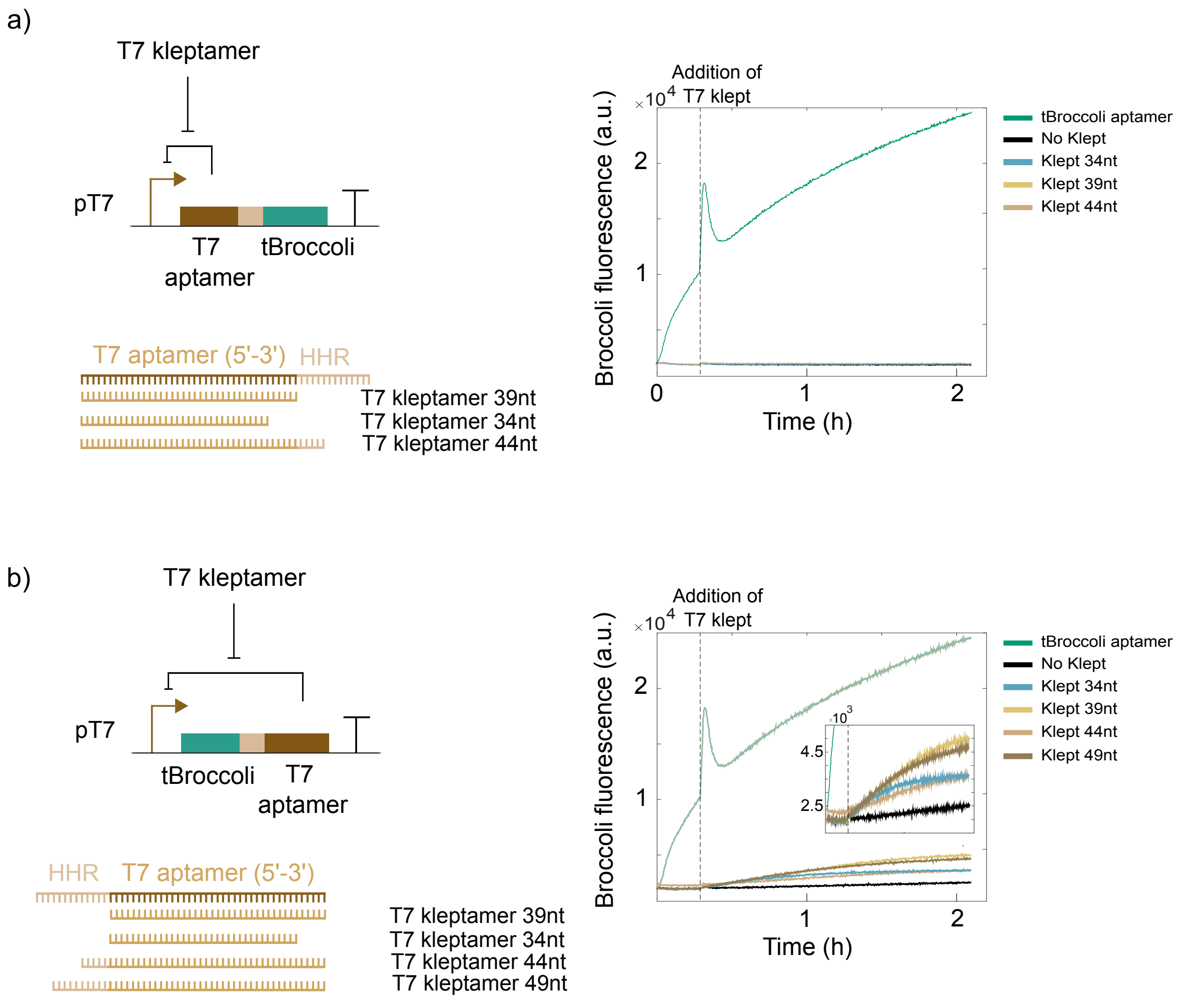

SI Figure 3: **Comparison of the performance of T7 cis-acting circuits with different architectures with their corresponding kleptamers.** **a)** Cis-acting circuits where the transcription of the T7 inhibitory aptamer is followed by the tBroccoli fluorescent aptamer via T7 promoter. T7 kleptamers covering the full and partial inhibitory aptamer were used as well as with a short toehold downstream. **b)** T7 cis-acting circuits where the transcription of the tBroccoli aptamer is followed by the SP6 inhibitory aptamer. Green lines indicates the expression of only Malachite Green fluorescence signal showing the cost of expressing more elements in the same transcript. Spikes in fluorescence near the times indicated by the vertical lines are due to injection-induced temperature decrease. Solid lines correspond to a single replicate to facilitate the visualization of the results. Technical replicates can be found in SI Figure 6.

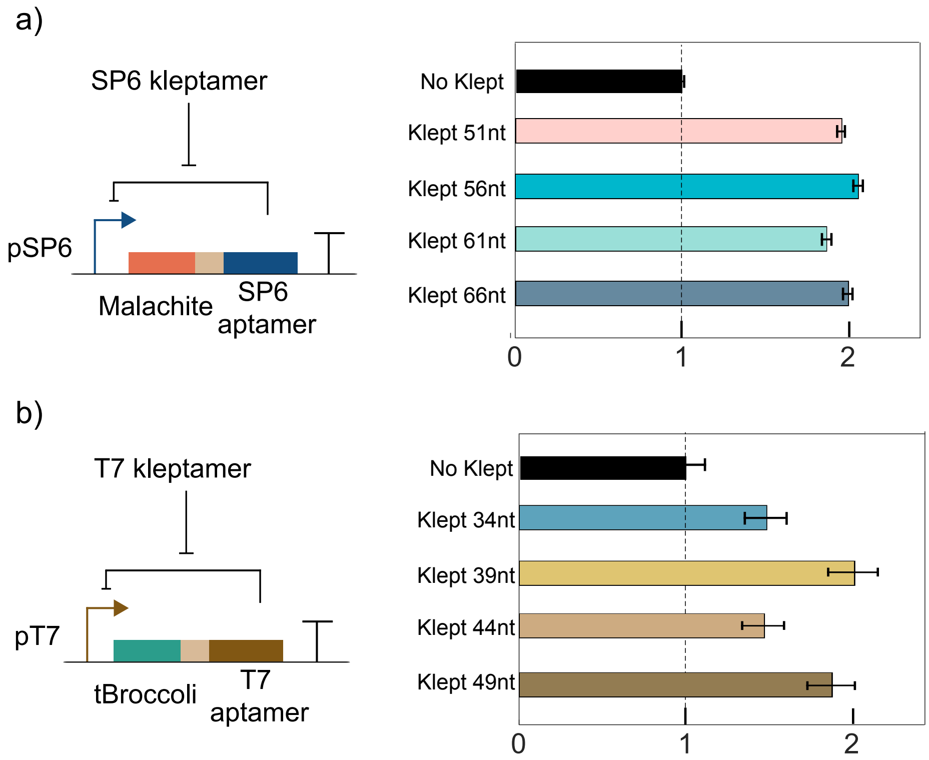

SI Figure 4: Normalization signal from the endpoints of each sample compared to the negative control for **a)** SP6 cis-acting circuits and **b)** T7 cis-acting circuit. The average of the fluorescence signal across the last 10 time points (less than 10 minutes) was performed in each sample. This value was normalized to the negative control to obtain the fold change. The error bars were calculated following the propagation error formula.

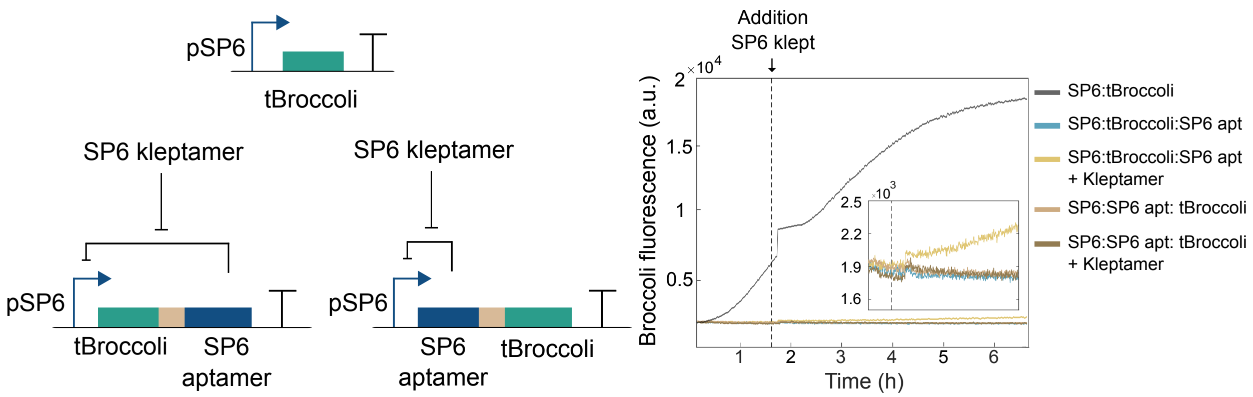

SI Figure 5: **SP6 Cis-acting circuit with tBroccoli as reporter.** SP6 promoter transcribes tBroccoli and tBroccoli along with the SP6 inhibitory aptamer in both positions. Only when tBroccoli was expressed before the inhibitory aptamer, it was possible to observe an increase in tBroccoli signal after adding SP6 kleptamer. The spike in fluorescence near the times indicated by the vertical lines are due to injection-induced temperature decrease. Solid lines correspond to a single replicate.

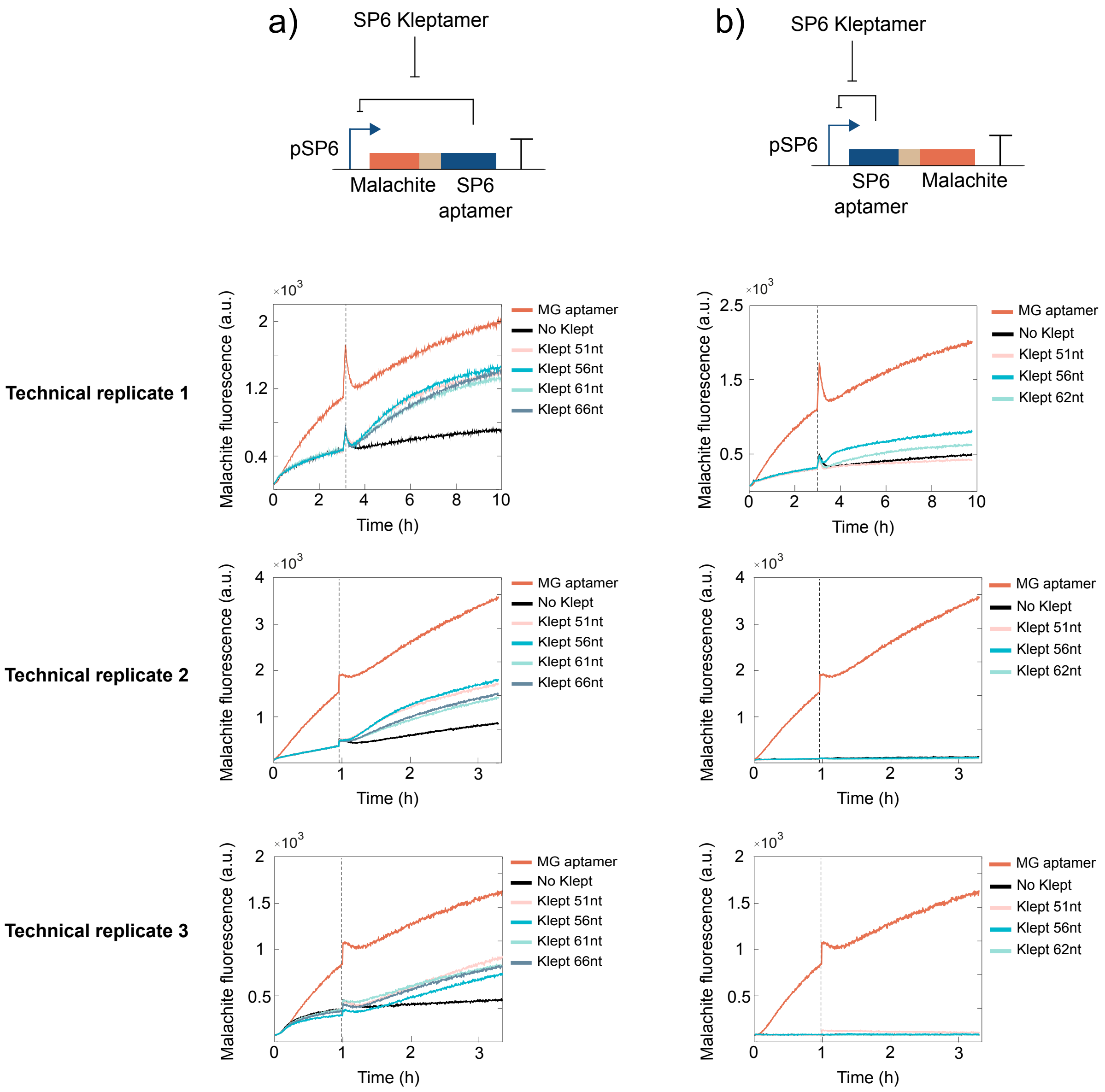

SI Figure 6: **Technical replicates of SP6 cis-acting circuits.** **a)** SP6 cis-acting circuit with the fluorescence aptamer being expressed before the inhibitory aptamer. **b)** SP6 cis-acting circuit with the inhibitory aptamer being expressed before the fluorescence aptamer. The spikes in fluorescence near the times indicated by the vertical lines are due to injection-induced temperature decrease.

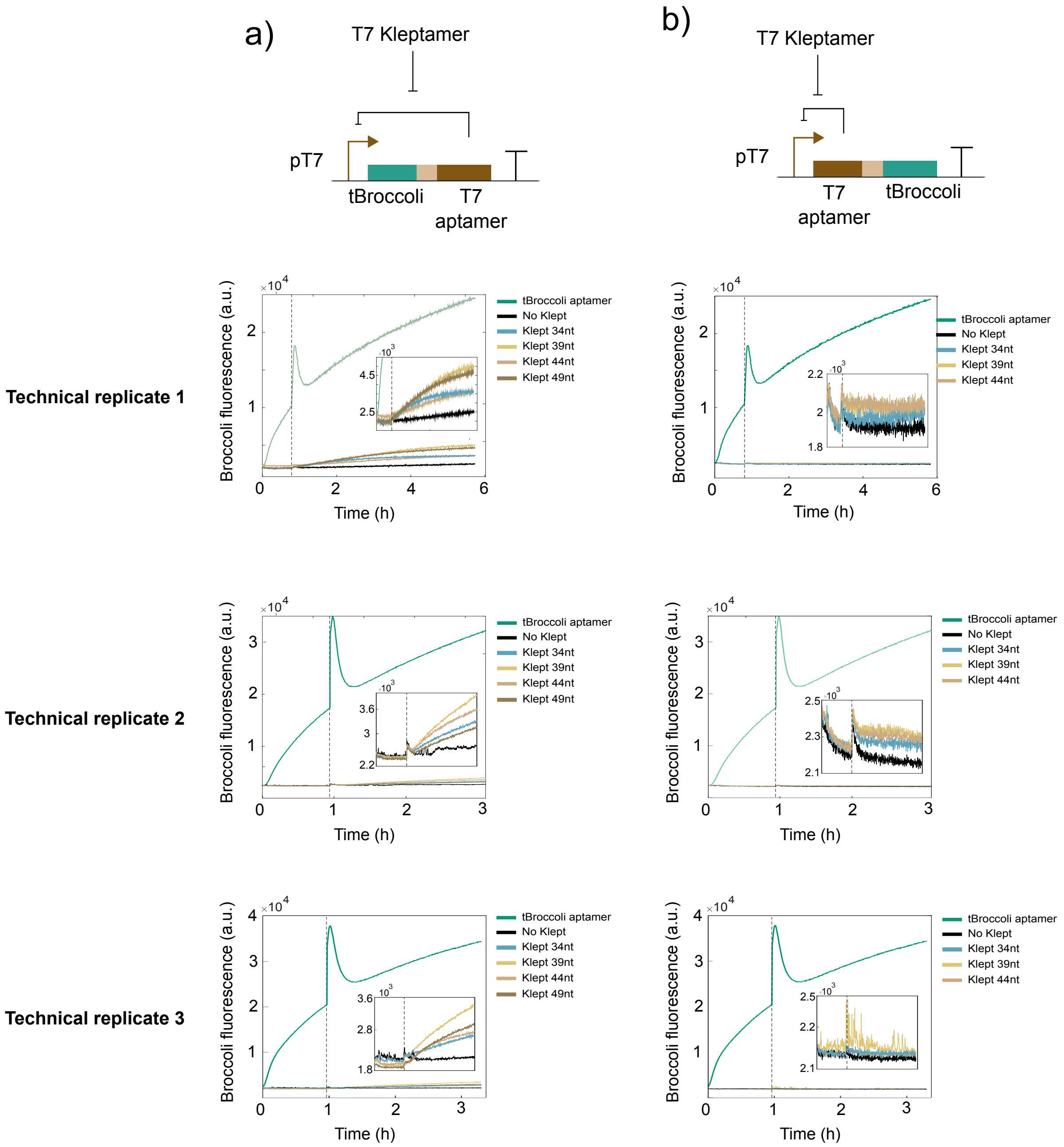

SI Figure 7: **Technical replicates of T7 cis-acting circuits. a)** T7 cis-acting circuit with the fluorescence aptamer being expressed before the inhibitory aptamer. **b)** T7 cis-acting circuit with the inhibitory aptamer being expressed before the fluorescence aptamer. The spikes in fluorescence near the times indicated by the vertical lines are due to injection-induced temperature decrease.

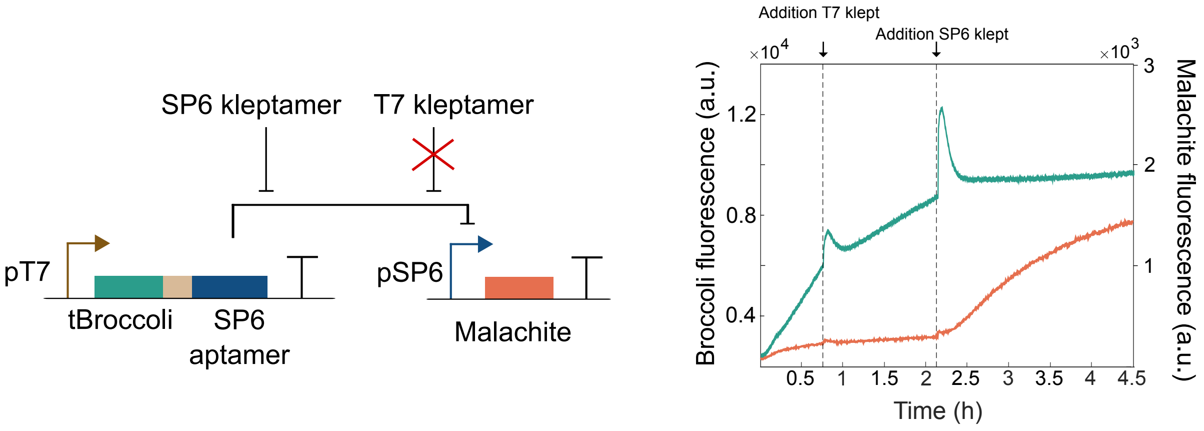

SI Figure 8: **Cross-talk between DNA kleptamers tested with SP6 trans-acting circuit**. The expression of SP6 inhibitory aptamer represses the production of Malachite Green aptamer. Malachite Green aptamer was still repressed after adding the DNA kleptamer against the T7 inhibitory aptamer (first black vertical dashed line). Only after the addition of the adequate DNA kleptamer, the SP6 RNAP was able to start the transcription of this fluorescent aptamer. Solid lines correspond to a single replicate.

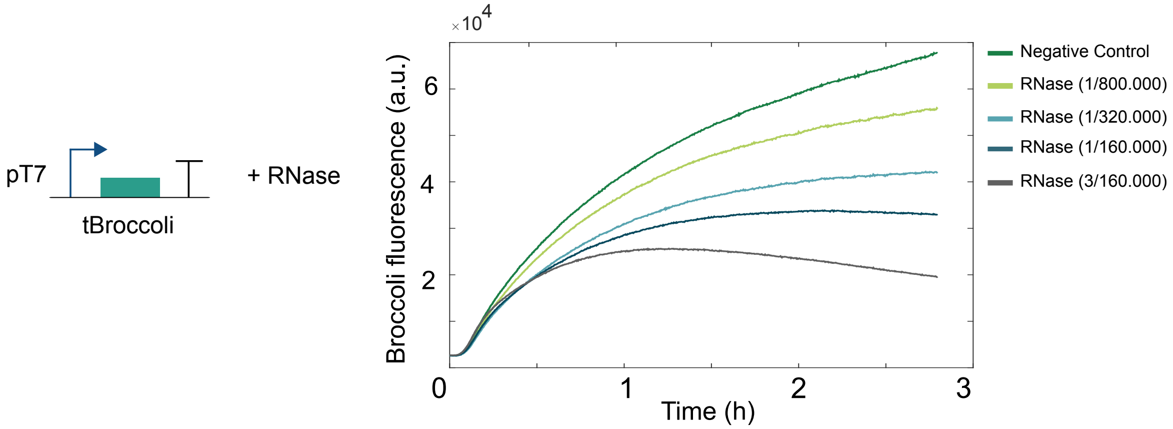

SI Figure 9: **Characterization of RNase molecules using tBroccoli aptamer.** Different concentrations of RNAse molecules were tested to obtain a balance between production and degradation of RNA molecules. Negative control represents the fluorescent aptamer without RNAse. Dilutions were calculated based on the commercial RNAse (Methods). Solid lines correspond to a single replicate.

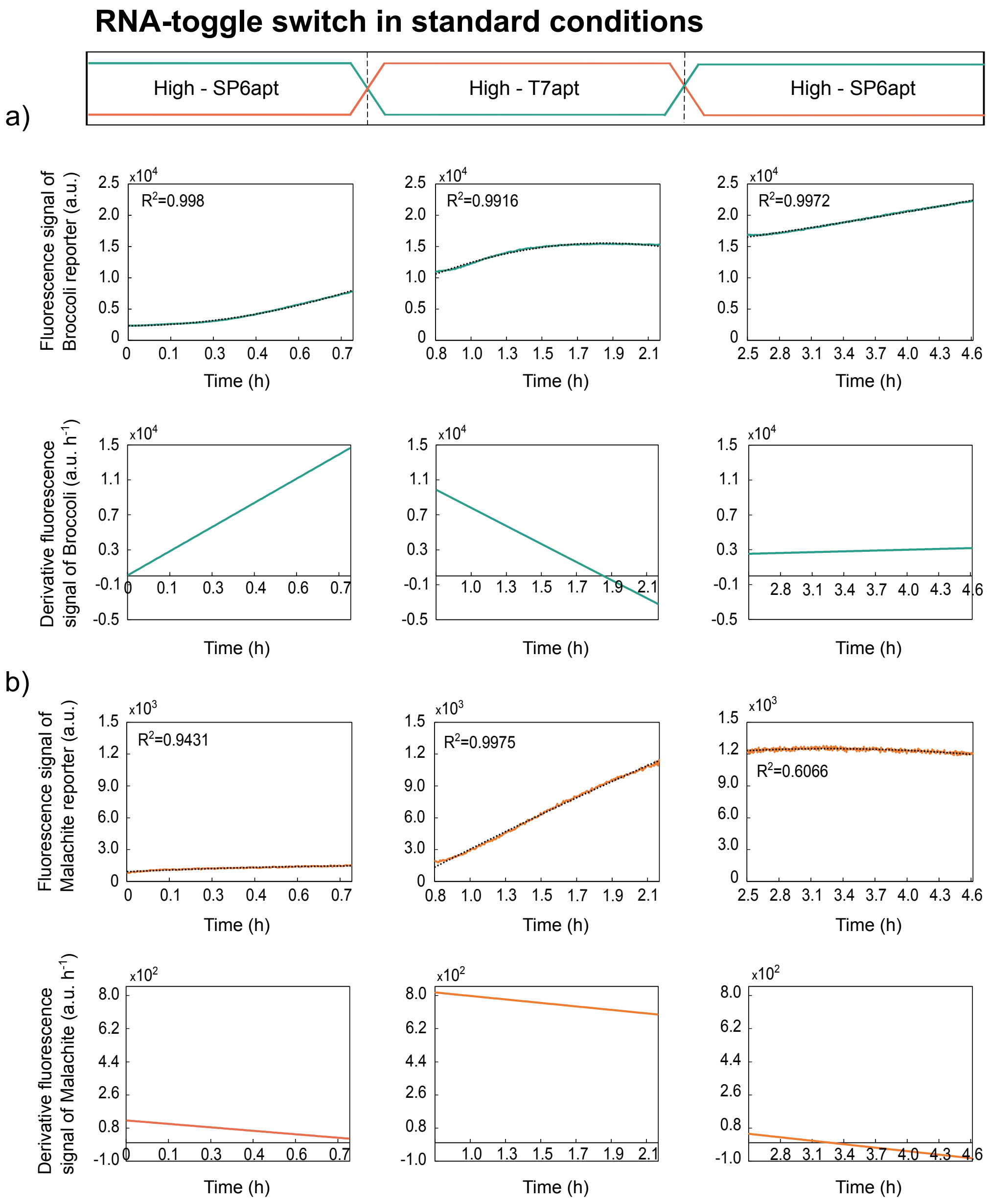

SI Figure 10: **Production rate of both RNA fluorescent species in the RNA-based toggle switch in standard conditions.** a) Broccoli fluorescent signal over time was fitted to a quadratic equation for each section (upper plots and green lines) and derivates of these fluorescent signals were calculated to present the production rate of Broccoli fluorescent aptamer (lower plots and green lines). b) Malachite fluorescent signal over time was fitted to a quadratic equation for each section (upper plots and orange lines) and derivates of these fluorescent signals were calculated to present the production rate of Malachite fluorescent aptamer (lower plots and orange lines). The signal pulse period was not included in this analysis since it is not representative of the behaviour of the circuit.

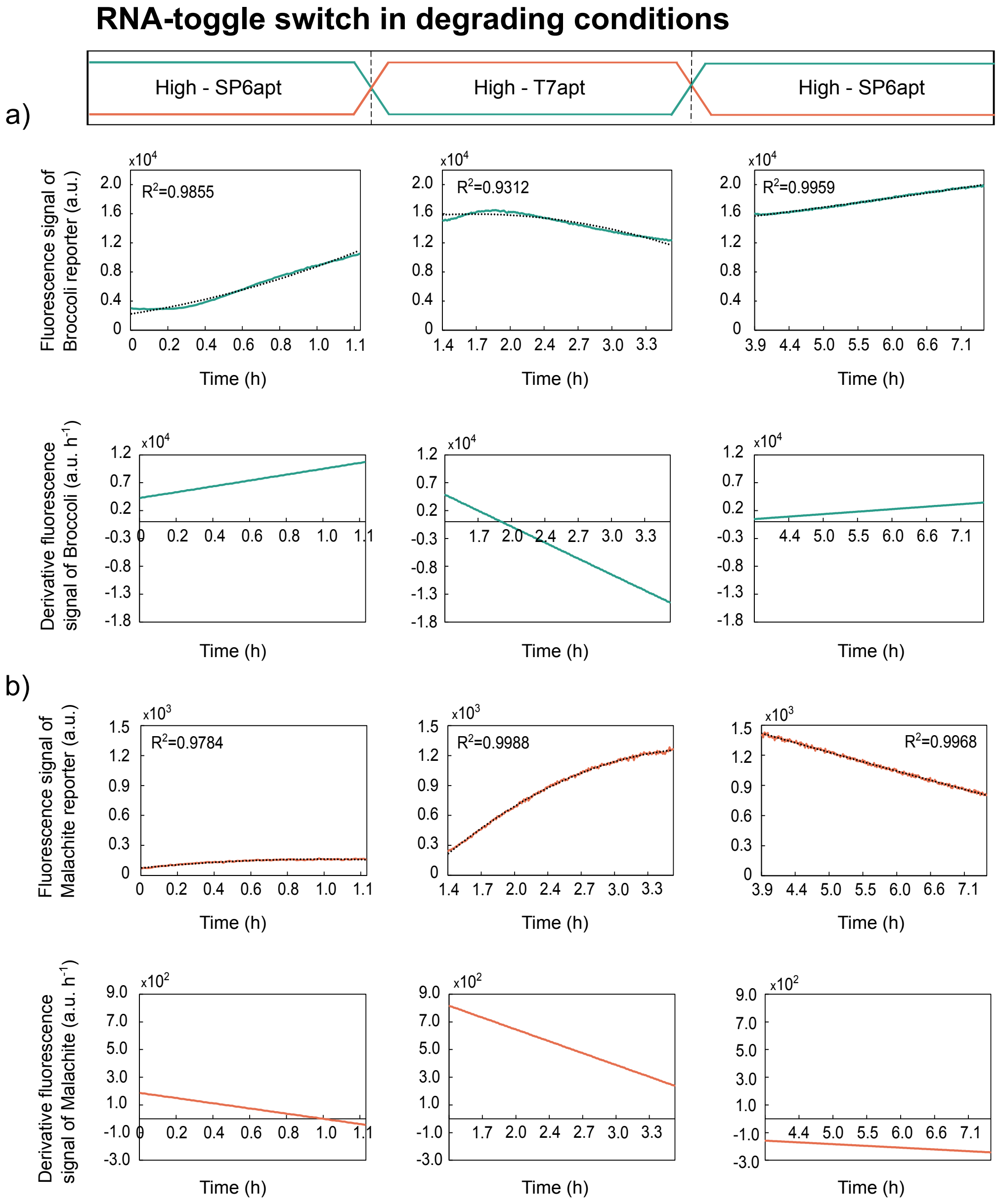

SI Figure 11: **Production rate of both RNA fluorescent species in the RNA-based toggle switch in degrading conditions (Figure 4c).** a) Broccoli fluorescent signal over time was fitted to a quadratic equation for each section (upper plots and green lines) and derivates of these fluorescent signals were calculated to present the production rate of Broccoli fluorescent aptamer (lower plots and green lines). b) Malachite fluorescent signal over time was fitted to a quadratic equation for each section (upper plots and orange lines) and derivates of these fluorescent signals were calculated to present the production rate of Malachite fluorescent aptamer (lower plots and orange lines). The signal pulse period was not included in this analysis since it is not representative of the behaviour of the circuit.

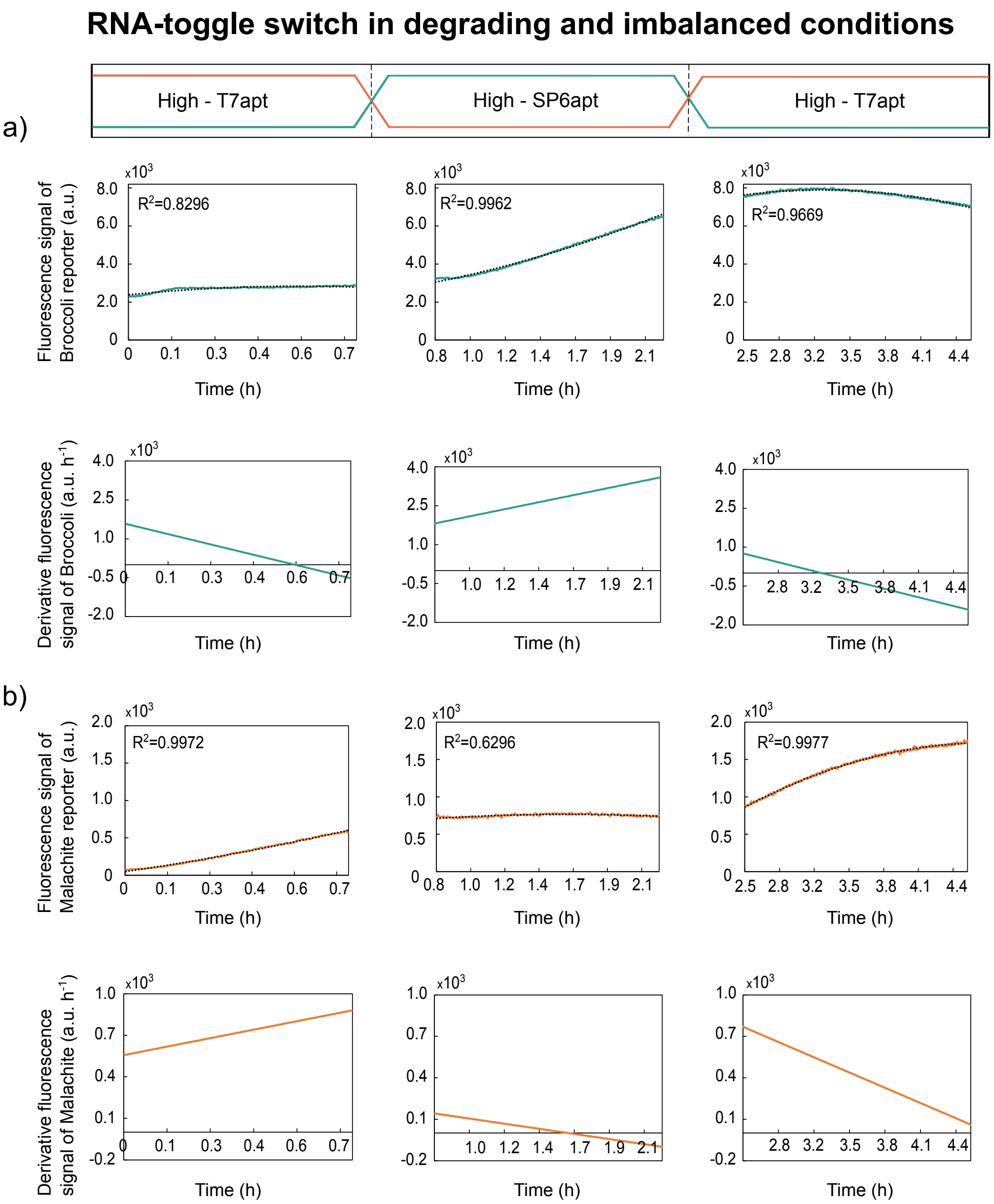

SI Figure 12: **Production rate of both RNA fluorescent species in the RNA-based toggle switch in degrading and imbalanced conditions (Figure 4d).** a) Broccoli fluorescent signal over time was fitted to a quadratic equation for each section (upper plots and green lines) and derivates of these fluorescent signals were calculated to present the production rate of Broccoli fluorescent aptamer (lower plots and green lines). b) Malachite fluorescent signal over time was fitted to a quadratic equation for each section (upper plots and orange lines) and derivates of these fluorescent signals were calculated to present the production rate of Malachite fluorescent aptamer (lower plots and orange lines). The signal pulse period was not included in this analysis since it is not representative of the behaviour of the circuit.

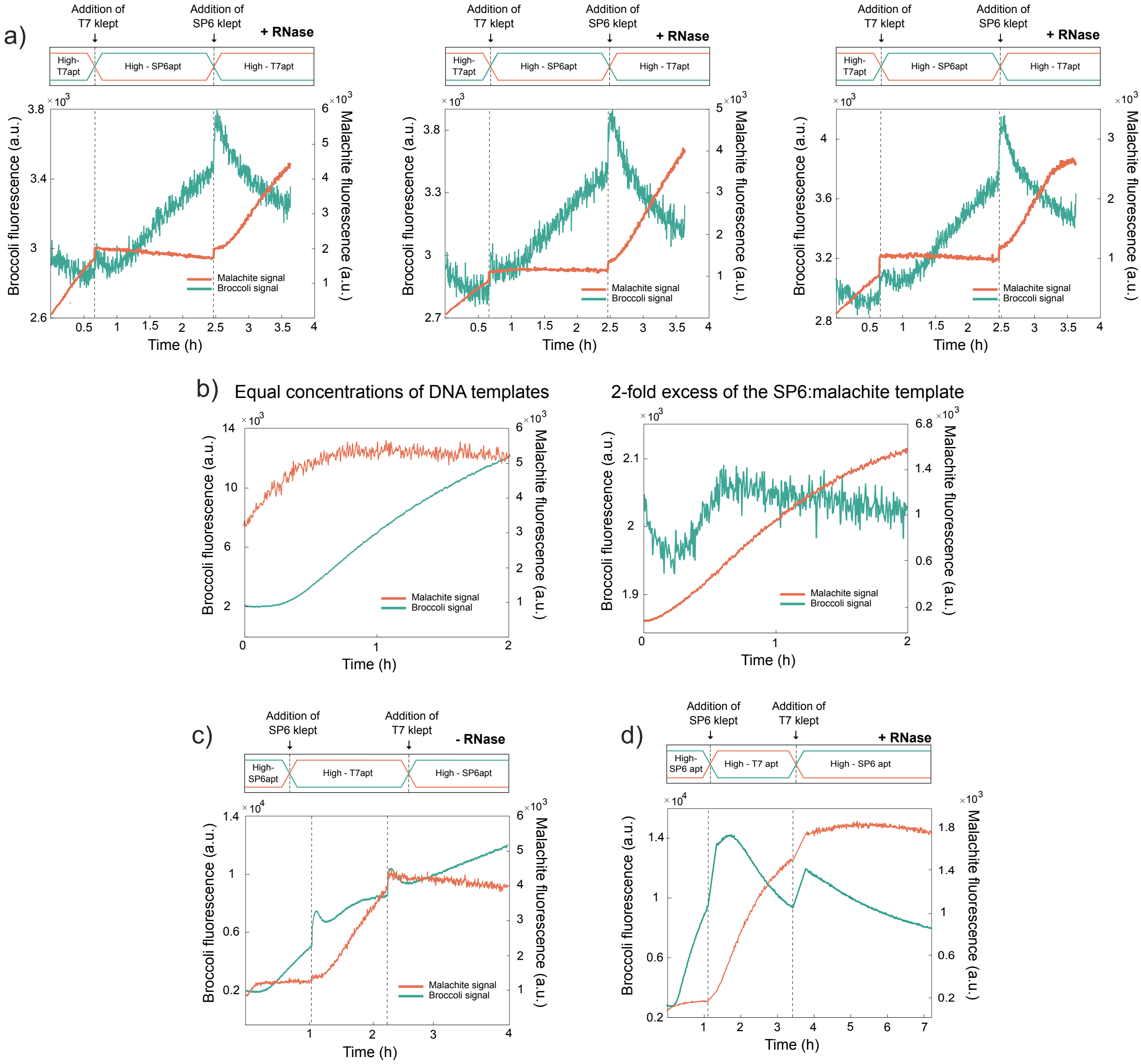

SI Figure 13: **Replicates of the RNA-based Toggle Switch**. **a)** Replicates of the toggle switch with RNA-degrading conditions and an imbalance in the amount of DNA templates. **b)** Supporting experiment to prove control over the system in RNA-degrading conditions. When both DNA templates of the toggle switch are present in equal concentration (200 ng per template), the template with the stronger promoter (i.e. T7 promoter) dominates the reaction. However, a 2-fold excess of the DNA template with the SP6 promoter400 ng) is sufficient for this promoter to dominate in the first stage of the reaction. Different scales were used in these graphs. **c)** Technical replicate of the toggle switch in standard conditions. **d)** Technical replicate of the toggle switch in degrading conditions.

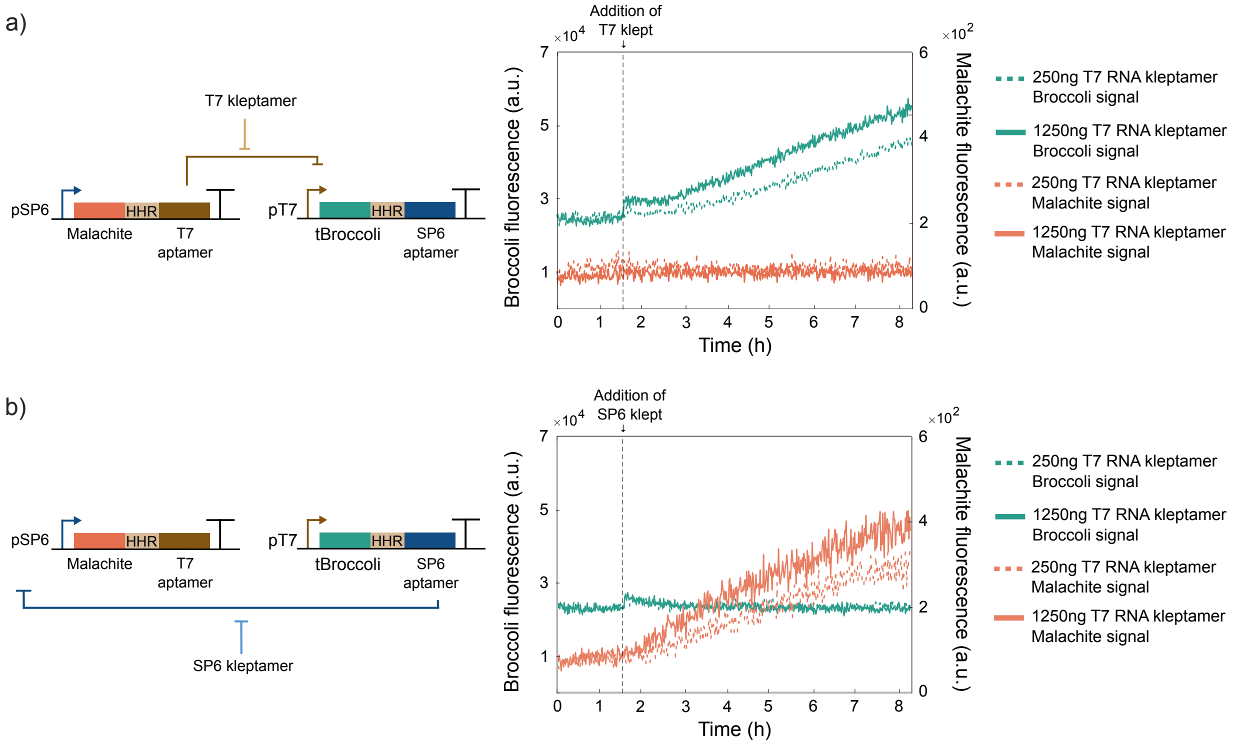

SI Figure 14: **Performance of trans-acting circuits using RNA kleptamers. a)** State A of the RNA-based toggle switch where the expression of T7 inhibitory aptamer is repressing tBroccoli. The addition of T7 RNA kleptamer at 250 ng and 1250 ng increased tBroccoli fluorescence signal. **b)** State B of the RNA-based toggle switch where the expression of SP6 inhibitory aptamer is repressing Malachite Green aptamer. The addition of SP6 RNA kleptamer at 250 ng and 1250 ng increased Malachite Green fluorescence signal. Spikes in fluorescence are due to injection-induced temperature decrease. Solid lines correspond to a single replicate.
